# IAA-miR164a-NAC100L1 module mediates symbiotic incompatibility of cucumber/pumpkin grafted seedlings through regulating callose deposition

**DOI:** 10.1093/hr/uhad287

**Published:** 2023-12-29

**Authors:** Mingzhu Yuan, Tong Jin, Jianqiang Wu, Lan Li, Guangling Chen, Jiaqi Chen, Yu Wang, Jin Sun

**Affiliations:** College of Horticulture, Nanjing Agricultural University, Nanjing 210095, China; College of Horticulture, Nanjing Agricultural University, Nanjing 210095, China; College of Horticulture, Nanjing Agricultural University, Nanjing 210095, China; College of Horticulture, Nanjing Agricultural University, Nanjing 210095, China; College of Horticulture, Nanjing Agricultural University, Nanjing 210095, China; College of Horticulture, Nanjing Agricultural University, Nanjing 210095, China; College of Horticulture, Nanjing Agricultural University, Nanjing 210095, China; College of Horticulture, Nanjing Agricultural University, Nanjing 210095, China

## Abstract

Grafting is one of the key technologies to overcome the obstacles of continuous cropping, and improve crop yield and quality. However, the symbiotic incompatibility between rootstock and scion affects the normal growth and development of grafted seedlings after survival. The specific molecular regulation mechanism of graft incompatibility is still largely unclear. In this study, we found that the IAA-miR164a-NAC100L1 module induced callose deposition to mediate the symbiotic incompatibility of cucumber/pumpkin grafted seedlings. The incompatible combination (IG) grafting interface accumulated more callose, and the activity of callose synthase (CmCalS1) and IAA content were significantly higher than in the compatible combination (CG). Treatment with IAA polar transport inhibitor in the root of the IG plants decreased CmCalS activity and callose content. Furthermore, IAA negatively regulated the expression of Cm-miR164a, which directly targeted cleavage of *CmNAC100L1*. Interestingly, CmNAC100L1 interacted with CmCalS1 to regulate its activity. Further analysis showed that the interaction between CmNAC100L1 and CmCalS1 increased the activity of CmCalS1 in the IG plants but decreased it in the CG plants. Point mutation analysis revealed that threonine at the 57th position of CmCalS1 protein played a critical role to maintain its enzyme activity in the incompatible rootstock. Thus, IAA inhibited the expression of Cm-miR164a to elevate the expression of *CmNAC100L1*, which promoted CmNAC100L1 interaction with CmCalS1 to enhance CmCalS1 activity, resulting in callose deposition and symbiotic incompatibility of cucumber/pumpkin grafted seedlings.

## Introduction

Cucumber (*Cucumis sativus* L.) is one of the main vegetable crops under protected cultivation in China, and the total yield of cucumber is 56.24 million tons [1]. In recent years, soil continuous cropping obstacles have been serious in protected cultivation, while cucumber roots are fragile, and are sensitive to continuous cropping obstacles and unsuitable cultivation conditions [2]. Grafting is often used in production to overcome cucumber continuous cropping obstacles and various biological and abiotic stresses [3–5]. Symbiotic compatible grafting promotes plant growth and increases crop yield, while the growth potential of symbiotic incompatible grafting is weak, resulting in agricultural yield reduction and crop quality decline [6, 7]. Pumpkin is widely used as the rootstock for cucumber grafting cultivation. However, symbiotic incompatibility also occurs during pumpkin and cucumber grafting [8, 9], which has a negative impact on the growth and development of grafted seedlings. Therefore, overcoming the symbiotic incompatibility of grafting is the key technical problem to achieve the goal of grafting.

Callose (β-1,3-glucan), is commonly found in many plant tissues [10]. When plants suffer from biotic and abiotic stresses, callose is synthesized in a large amount at the plasmodesmata [11]. Different types of deposits, including callose, often appear at the interface after grafting. Frey *et al*. [12] used tomato for grafting, and found that there was a large amount of callose deposition at the interface of the failed combination. Furthermore, Xiong *et al*. [13] also found a large amount of callose deposition at the interface of graft-incompatible combinations in melon grafting combinations. It can be seen that callose deposition is related to graft compatibility, but the molecular mechanism of callose deposition during graft healing and how to mediate grafting incompatibility are still unknown. In addition, the healing process of grafted plants also involves complex hormone signaling [14]. Transcriptome analysis of citrus grafted compatible and incompatible combinations revealed that several genes associated with auxin induction and response pathways were differentially expressed [15]. Ji *et al*. [16] found that gene expression associated with auxin signaling biosynthesis was delayed during the healing process of incompatibly grafted plants in pears. It can be seen that auxin plays an important role in the grafting healing process, but there is no more detailed information on molecular mechanisms regulating grafting incompatibility.

**Figure 1:**
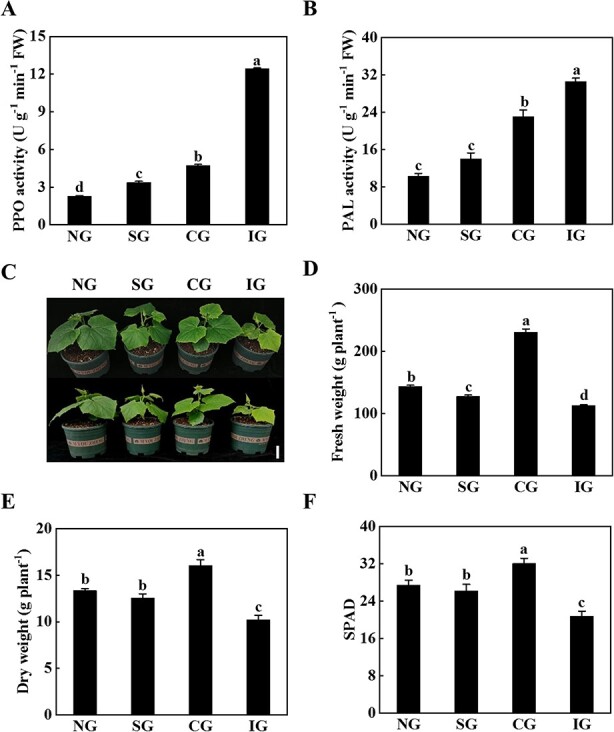
Symbiotic incompatibility inhibited the growth of cucumber/pumpkin grafted seedlings. **A** PPO activity. **B** PAL activity. **C** Plant phenotype 25 days after grafting. Scale bar: 10 cm. **D** Fresh weight. **E** Dry weight. **F** SPAD value. Phenotype, PPO activity, PAL activity, and SPAD value were measured 25 days after grafting, and biomass was measured at 70 days. Data represent mean ± standard deviation (*n* = 3). According to Tukey’s test, means with the same letter did not differ significantly at *P* < 0.05. FW, fresh weight; NG, non-grafted; SG, self-grafted; CG, compatible combination; IG, incompatible combination.

A recent study has found that the expression profiles of miRNA will change after the deposition of callose [17]. Under aluminum stress, callose fluorescence was observed at the mature nodes of soybean, and the expression of miR164 and miR396 increased [18]. Cassava stem infected by cassava anthracnose will cause callose deposition [19]. Interestingly, the presence of mes-miR156 and mes-miR164 was observed in the recovered fungal cells [19]. Wang *et al*. [20] found that the expression of osa-miR164a decreased in the early and late stages of rice blast Guy11 infection, accompanied by more production of reactive oxygen species and callose deposition, as well as the upregulation of defense-related genes. These results indicate that callose deposition may be regulated by miRNA, but the specific molecular regulation mechanism between them has not been revealed.

With the continuous deepening of graft research, various omics techniques have been widely used to screen genes and miRNAs related to graft incompatibility [15, 21]. In our previous study, we screened 60 miRNAs related to graft incompatibility through RNA sequencing technology [9]. Among them, miR164 was differentially expressed in compatible and incompatible combinations, suggesting that miR164 might be closely related to cucumber/pumpkin graft incompatibility [9]. However, it is not clear whether miR164 causes incompatibility by promoting callose deposition at the grafting wound. Therefore, this study clarified that the amount of callose deposition at the graft interface affected graft compatibility through the analysis of phenotypic and physiological data. Further molecular experiments showed that IAA suppressed the expression of miR164a. The rapid amplification of cDNA terminal mediated by 5′ RNA ligase (5′-RLM–RACE) and tobacco transient transformation experiments showed that miR164a targeted the *NAC100L1* gene. Moreover, NAC100L1 interacted with callose synthase 1 (CalS1) and enhanced its enzyme activity in graft-incompatible combination, which promoted the accumulation of callose deposition, resulting in a graft incompatibility reaction. These findings reveal the important role of IAA in rootstocks in mediating graft incompatibility by regulating the activity of CalS, and clarify the molecular mechanism of graft incompatibility, which is of great significance in enriching the theory of horticultural plant grafting.

## Results

### Physiological indexes reflected the compatibility/incompatibility of grafted seedlings

Polyphenol oxidase (PPO) and phenylalanine ammonialyase (PAL) activity, fresh weight, dry weight, and leaf relative chlorophyll content (SPAD) were analyzed 25 days after grafting in different grafting combinations, including non-grafted (NG), self-grafted (SG), compatible combination (CG, cucumber grafted onto figleaf gourd), and incompatible combination (IG, cucumber grafted onto ‘Dongyangshenli’). The activity of PPO and PAL at the graft junction of the IG plants was significantly increased compared with the activity of PPO and PAL in the NG, SG, and CG plants (Fig. 1A and B). Furthermore, the CG grafted seedlings grew well, but the IG grafted seedlings grew slowly, as indicated by decreased fresh weight, which decreased by 21.52, 11.61, and 51.09%, respectively, compared with the NG, SG, and CG plants (Fig. 1C and D). The CG plants had the highest dry weight, which was significantly higher than that of the IG plants (Fig. 1E). In addition, the IG seedlings showed the lowest SPAD value (Fig. 1F). These results indicated that the incompatible grafting combination significantly inhibited the growth of grafted seedlings.

### Incompatible grafting combination induced callose deposition

**Figure 2:**
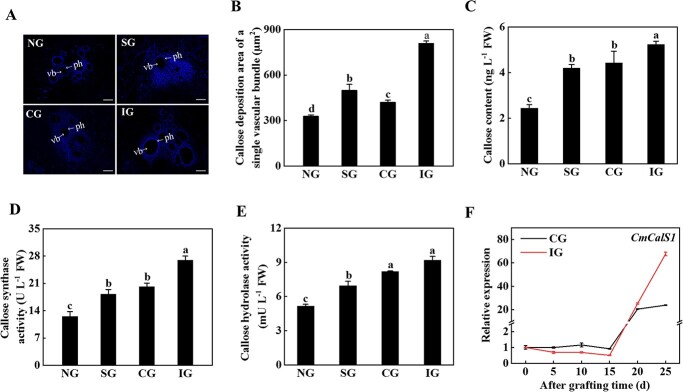
Symbiotic incompatibility induced callose deposition at the graft junction. **A** Twenty-five days after grafting, aniline blue staining was performed at the graft junction to observe the deposition of callose. Arrows indicate the phloem (ph) and the vascular bundles (vb). Scale bar: 100 μm. **B** Area of callose deposition of a single vascular bundle in **A**. **C** Callose content. **D** Callose synthase activity. **E** Callose hydrolase activity. **F** Expression of *CmCalS1*. Expression of the *CmCalS1* gene was analyzed by qPCR in the rootstock of the grafted junction of different compatible combinations after grafting. Data represent the mean ± standard deviation (*n* = 3). According to Tukey’s test, means with the same letter did not differ significantly at *P* < 0.05. FW, fresh weight; NG, non-grafted; SG, self-grafted; CG, compatible combination; IG, incompatible combination.

To investigate whether callose mediated graft incompatibility, the content of callose and the activity of CalS and callose hydrolase at the graft junction were measured. The results showed that callose was mainly deposited in phloem in the junction of the grafting wound (Fig. 2A). The area of callose deposition of single vascular bundles at the wound healing site in the IG seedlings was 1.92-fold of the area of callose deposition in the CG seedlings (Fig. 2B). There were significant differences in the content of callose and the activity of CalS and callose hydrolase in the rootstock of the grafted junction in different graft combinations (Fig. 2C–E). The activity of CalS and callose hydrolase in the IG plants was the highest, 25.13 and 10.80% higher than the level in the CG plants, respectively (Fig. 2D and E).

To further analyze the role of CalS in callose deposition, the expression levels of *CalS* genes in pumpkin were analyzed. In the first 15 days, the expression of *CmCalS1* in the CG and IG plants was slightly changed, but its level dramatically increased at the 20th and 25th days (Fig. 2F). Although the expression of *CmCalS1* reached the highest level at 25 days, its level in the IG plants was 2.83-fold of that in the CG plants (Fig. 2F). The expression of *CmCalS9* in the CG plants decreased gradually with the prolongation of grafting time, and decreased to the lowest value on the 25th day after grafting, while the expression of *CmCalS9* in the IG plants decreased first and then increased, and reached the peak on the 20th day after grafting (Supplementary Data Fig. S1A). There was no significant difference in the expression of *CmCalS11* in the CG plants with the extension of grafting time, and there was also no significant difference in the expression of *CmCalS11* in the first 15 days in the IG plants, but expression gradually increased after the 20th day; at the 25th day it was 1.87-fold higher than that at 0 days (Supplementary Data Fig. S1B). These results indicated that in the later stage of grafting, i.e. when the grafted seedlings were fully viable and began to grow (symbiotic compatibility stage), the expression of *CmCalS1* in the IG plants increased significantly, resulting in the deposition of callose.

### Effects of IAA on callose content and callose synthase activity

**Figure 3:**
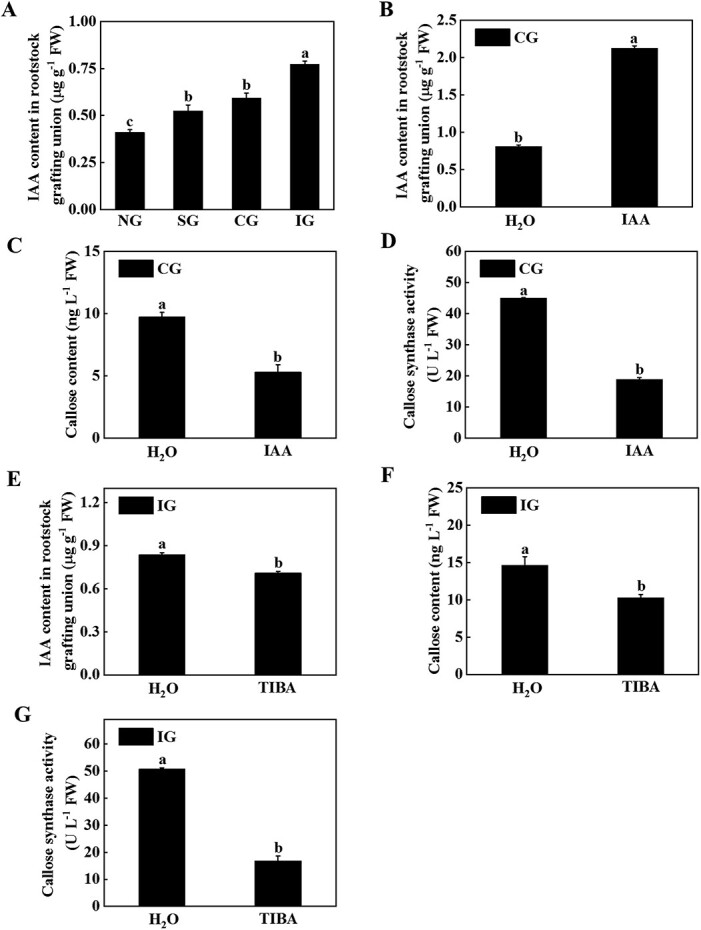
Effects of IAA on callose content and callose synthase activity. **A** IAA content in the rootstock of the grafted junction of different grafting combinations 25 days after grafting. **B** Effects of exogenous treatment with IAA in the root of compatible combination (CG) on IAA content in the rootstock of the grafted junction. **C** Effects of exogenous treatment with IAA in the root of CG on callose content in the rootstock of the grafted junction. **D** Effects of exogenous treatment with IAA in the root of CG on callose synthase activity in the rootstock of the grafted junction. **E** Effects of exogenous treatment with TIBA in the root of the incompatible combination (IG) on IAA content in the rootstock of the grafted junction. **F** Effects of exogenous treatment with TIBA in the root of IG on callose content in the rootstock of the grafted junction. **G** Effects of exogenous treatment with TIBA in the root of IG on callose synthase activity in the rootstock of the grafted junction. Nineteen days after grafting, 10 μM IAA or TIBA was applied and the IAA content, callose content, and callose synthase activity were measured after treatment for 6 days. Data represent the mean ± standard deviation (*n* = 3). According to Tukey’s test, means with the same letter did not differ significantly at *P* < 0.05. FW, fresh weight; NG, non-grafted; SG, self-grafted.

IAA has been shown to regulate the development of callose [22]. In order to clarify the role of IAA in different grafting combinations, we measured IAA content in the rootstock of grafted junctions in different graft combinations (Fig. 3A). The results showed that grafting significantly induced an increase in IAA, but this trend was more obvious in the IG plants (Fig. 3A). These results indicated that IAA might mediate the deposition of callose. To further investigate the role of IAA in callose deposition, exogenous IAA or a polar transport inhibitor of IAA, 2,3,5-triiodobenzoic acid (TIBA), was used to treat the root of the CG or IG plants, respectively. IAA treatment of the CG combined roots was found to significantly increase IAA content, reduce callose content and CalS activity (Fig. 3B–D). After application of TIBA to the IG combined roots it was found that IAA content, callose content, and CalS activity were significantly reduced (Fig. 3E–G). Furthermore, we observed the plant phenotype and measured the growth indicators of the grafted plants treated with IAA or TIBA. Treatment with IAA or TIBA of the CG or IG combined roots promoted the growth of grafted plants (Supplementary Data Figs S2 andS3). Plant height, stem diameter, fresh weight, dry weight, and SPAD of TIBA-treated plants were significantly higher than those in the control plants (Supplementary Data Fig. S3). Thus, different concentrations of IAA in the CG and IG combinations affect symbiotic compatibility by affecting callose content.

### IAA negatively regulates the expression of Cm-miR164a

**Figure 4:**
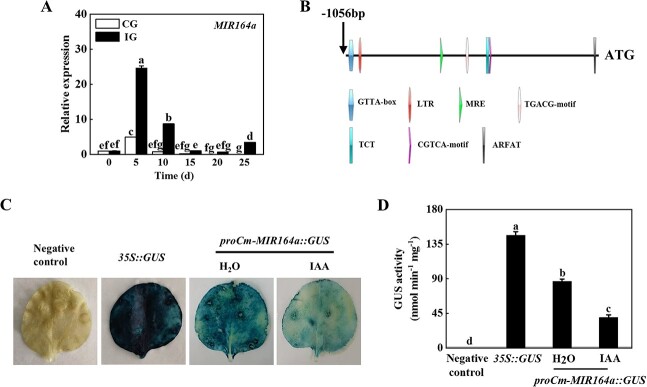
IAA negatively regulated Cm-miR164a expression. **A** Expression of Cm-miR164a in the compatible combination (CG) and incompatible combination (IG). **B** Analysis of *cis*-acting elements of the *Cm-MIR164a* promoter. **C**, **D** Transient GUS expression in tobacco leaves showed that IAA inhibited the expression of Cm-miR164a. *Agrobacterium tumefaciens* containing the indicated plasmid was injected into tobacco leaves, and the leaves were treated with 100 μM IAA 1 day after injection. GUS staining and activity were determined 2 days after injection. Data represent the mean ± standard deviation (*n* = 3). According to Tukey’s test, means with the same letter did not differ significantly at *P* < 0.05.

The above experiments showed that IAA might mediate the symbiotic incompatibility response to grafting by regulating the deposition of callose, indicating that rootstock IAA played an important role in regulating the cucumber/pumpkin grafted seedling incompatibility response. Furthermore, our previous research suggests that Cm-miR164a may also be closely related to cucumber/pumpkin grafting incompatibility [9]. In order to test whether IAA and Cm-miR164a co-regulated the compatibility of cucumber/pumpkin grafted seedlings, we analyzed the expression of Cm-miR164a in the rootstock stem of the graft junction in CG and IG plants. Within 25 days of grafting, the expression of Cm-miR164a increased, and the peak was reached at the fifth day after grafting; expression was 23.62 times higher than that at 0 days in the IG plants (Fig. 4A). The expression of Cm-miR164a in the CG plants first increased and then decreased, peaked at the fifth day after grafting, and decreased to the lowest value on the 20th day after grafting, when it had decreased by 36.72% compared with its level at 0 days (Fig. 4A), indicating that Cm-miR164a responded to grafting and played different roles in grafted seedlings with different degrees of symbiotic compatibility. In order to further clarify the regulatory effect of IAA on Cm-miR164a, the upstream promoter sequence of pumpkin Cm-miR164a was obtained according to the NCBI database, and analyzed using the PlantCARE and PLACE online databases. Bioinformatics analysis predicted the presence of *cis*-acting elements in the *Cm-MIR164a* promoter, including the GATA-box, MRE, TGACG-motif, and ARFAT, where ARFAT is an auxin-responsive element (Fig. 4B). The *CaMV35S* promoter in the pBI121 vector containing the GUS reporter was replaced with the *Cm-MIR164a* promoter, and the GUS staining experiment was conducted using a tobacco transient transformation system. Tobacco leaves injected with *Cm-MIR164a* promoter vector were stained lighter after IAA treatment compared with the positive control and water-treated tobacco leaves (Fig. 4C). Consistent with the results of staining, the GUS activity of tobacco leaves treated with IAA was significantly lower than that of the positive control and water-treated leaves (Fig. 4D). Furthermore, the expression level of Cm-miR164a did indeed decrease after application of IAA, indicating that IAA inhibited the expression of *Cm-MIR164a* (Supplementary Data Fig. S4).

### Cm-miR164a negatively regulates the *CmNAC100L1* gene

**Figure 5:**
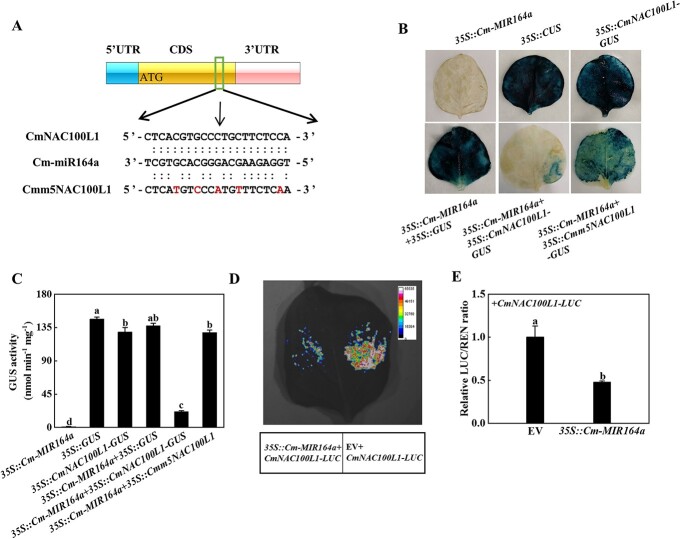
Pumpkin *CmNAC100L1* was the target gene of Cm-miR164a. **A** 5′-RLM-RACE verified the presence of a cleavage site in *CmNAC100L1*, and the cleavage site is indicated by a black arrow. **B**, **C** GUS staining experiments verified Cm-miR164a cleavage of *CmNAC100L1*. *Agrobacterium tumefaciens* containing the indicated plasmid was injected into tobacco leaves, and GUS staining and activity were measured 2 days after injection. **D**, **E** Dual luciferase assays verified the interaction between *CmNAC100L1* and Cm-miR164a. *Agrobacterium tumefaciens* containing the above plasmids was injected into tobacco leaves, which were analyzed 2 days after injection. Data represent the mean ± standard deviation (*n* = 3). According to Tukey’s test, means with the same letter did not differ significantly at *P* < 0.05. EV, empty vector.

Several members of the NAC-containing domain family play a healing-related role during grafting, promoting the formation of lamina cells after injury [23, 24]. The most likely target genes of miR164a were screened and predicted using psRNATarget. As shown in Supplementary Data Fig. S5A, Cm-miR164a and *CmNAC100L1* were paired at positions 617–637, with an expectation value of 2.0 and a unpaired energy (UPE) value of 9.221. Cm-miR164a was paired with *CmNAC79* at positions 424–444, with an expectation value of 9.5 and a UPE value of 20.009 (Supplementary Data Fig. S5B). Cm-miR164a was paired with *CmNAC100L2* at positions 656–676 and 1754–1774, with an expectation value of 3.0 and a UPE value of 15.68 (Supplementary Data Fig. S5C). Within 25 days after grafting, the expression of *CmNAC79* decreased first and then increased, dropped to the lowest value at the 5th day after grafting, reached the highest level on the 25th day after grafting, and had an obvious negative regulatory relationship with the expression of miR164a (Supplementary Data Fig. S6A). The expression of *CmNAC100L1* in the CG plants continued to rise, peaking at the 25th day after grafting (Supplementary Data Fig. S6B). However, the expression value of *CmNAC100L1* in the IG plants continued to decrease, and it dropped to the lowest value on the 25th day after grafting, when it had decreased by 74.35% (Supplementary Data Fig. S6B). The expression of *CmNAC100L2* continued to decrease, and the lowest value occurred on the 25th day after grafting in the CG plants, when it had decreased by 92.54%, but its level in the IG plants was continuously higher than the level in the CG plants (Supplementary Data Fig. S6C). Compared with the CG plants, the expression of *CmNAC100L1* at each time point in the IG plants was significantly reduced (Supplementary Data Fig. S6B). To verify the target relationship of Cm-miR164a with *CmNAC100L1*, *CmNAC100L2*, and *CmNAC79*, 5′-RLM–RACE was used to locate Cm-miR164a-oriented cutting sites in these three genes. The Cm-miR164a directed cleavage site was only located in the 10th and 11th base pairs of the miR164a target site in *CmNAC100L1* coding sequence (CDS) (Fig. 5A). Comparison of the miR164a sequence of Cucurbitaceae showed that the same bases, A and G, existed between bases 10 and 11 at the 5′ end, and the miR164a mature sequence in Cucurbitaceae was highly conserved (Supplementary Data Fig. S7A). Furthermore, the cleavage sites of Cm-miR164a in *CmNAC100L1* of figleaf gourd and ‘Dongyangshenli’ were completely consistent (Supplementary Data Fig. S7B), which indicated that Cm-miR164a targeted *CmNAC100L1*. To investigate whether *CmNAC100L1* was the target of Cm-miR164a, the interaction of Cm-miR164a with *CmNAC100L1* was determined in tobacco leaves by luciferase and GUS staining experiments. Blue staining was not detected in leaves infiltrated with *35S::Cm-MIR164a*, but dark blue staining was detected in leaves injected with *35S::GUS* or 3*5S::CmNAC100L1*-*GUS* or *35S::Cm-MIR164a* and *35S::GUS* (Fig. 5B). The intensity of the blue color and the blue ­colored area in the leaves co-injected with *35S::Cm-MIR164a* and *35S::CmNAC100L1-GUS* drastically decreased, while the blue staining increased when *35S::Cm-MIR164a* and *35S::Cmm5NAC100L1-GUS* were co-transformed into tobacco leaves (Fig. 5B). The GUS activity of tobacco leaves infiltrated with *35S::Cm-MIR164a* and *35S::CmNAC100L1-GUS* vectors was significantly lower than in other combinations (Fig. 5C). The fluorescence signal was weaker in leaves co-injected with *35S::CmNAC100L1-LUC* and *35S::Cm-MIR164a* than with *35S::CmNAC100L1-LUC* and empty vector (Fig. 5D). In addition, the relative LUC/REN ratio of leaves co-inoculated with *35S::CmNAC100L1-LUC* and *35S::Cm-MIR164a* was significantly lower than that of the control (Fig. 5E). Interestingly, tissue expression pattern analysis of *CmNAC100L1* showed that *CmNAC100L1* was predominately expressed in plant stems (Supplementary Data Fig. S8). Therefore, Cm-miR164a targeted *CmNAC100L1* and negatively regulated the expression of *CmNAC100L1* at the post-transcriptional level.

### CmNAC100L1 interacts with CmCalS1 protein to mediate CmCalS1's activity

**Figure 6:**
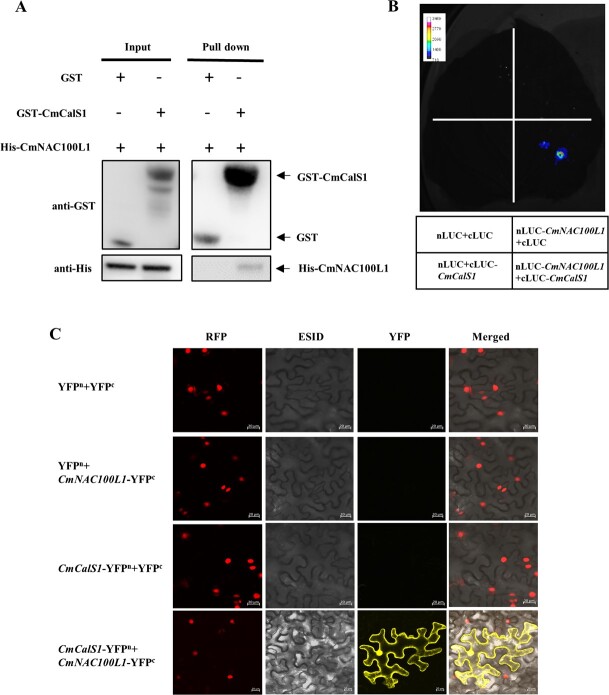
CmNAC100L1 interacted with CmCalS1. **A** Pull-down assay verification of CmNAC100L1 interaction with CmCalS1. **B** Firefly luciferase complementation assay verification of CmNAC100L1 interaction with CmCalS1. **C** BiFC assay verification of CmNAC100L1 interaction with CmCalS1. Scale bar: 20 μm.

Studies have shown that genes related to callose synthesis are regulated by MYB, ARF, and WRKY transcription factors [25, 26]. We tried to investigate whether CmNAC100L1 directly regulated the expression of *CmCalS1* using the yeast one-hybrid assay, but CmNAC100L1 failed to bind to the promoter of *CmCalS1* (Supplementary Data Fig. S9). Subcellular localization analysis revealed that the transcription factor CmNAC100L1 was located in the nucleus (Supplementary Data Fig. S10). At the same time, we also observed that CmCalS1 was distributed on both the cell membrane and the nucleus (Supplementary Data Fig. S10). Interestingly, CmNAC100L1 interacted with CmCalS1 (Fig. 6). As shown in Fig. 6A, His-CmNAC100L1 protein signal bound to GST-CmCalS1 could be detected with anti-His, but His-CmNAC100L1 protein signal co-incubated with GST could not be detected, indicating that CmCalS1 directly interacted with CmNAC100L1 *in vitro*. When nluc-*CmNAC100L1* and cluc-*CmCalS1* were co-transfected into tobacco leaves, the fluorescence signal was detected when sprayed with the substrate fluorescein, while the fluorescence signal was not detected when nluc-*CmNAC100L1* or cluc-*CmCalS1* was co-transfected with empty vector (Fig. 6B). In addition, YFP fluorescence was only detected on the cell membrane and nucleus after co-injection of *CmNAC100L1*-YFP^c^ with *CmCalS1*-YFP^n^, indicating that CmCalS1 and CmNAC100L1 bound to each other in the cell membrane and nucleus (Fig. 6C).

**Figure 7:**
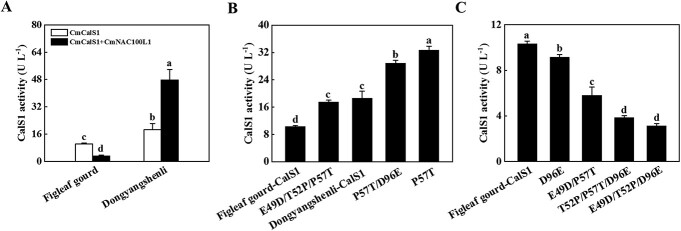
Effects of CmNAC100L1 and point mutation of CmCalS1 on its enzyme activity. **A** Comparison of effects of figleaf gourd and ‘Dongyangshenli’ CmNAC100L1 on CmCalS1 enzyme activity *in vitro*. CmNAC100L1 was loaded at 3.5 ng and CmCalS1 at 85 ng. **B** Point mutation increased CmCalS1 activity. **C** Point mutation decreased CmCalS1 activity.

To investigate the reason for the difference in CmCslS1 activity in the CG and IG plants, alignment of the nucleic acid and protein sequences of CmCalS1 from figleaf gourd and ‘Dongyangshenli’ was performed. There were small differences in the base sequence and protein sequence of *CmCalS1* gene in figleaf gourd and ‘Dongyangshenli’, which were manifested as two synonymous mutations and four non-synonymous mutations (Supplementary Data Fig. S11). Synonymous replacement of A with G at 144 bp from the start codon and non­synonymous replacement of T with G at 147 bp, resulted in the conversion of the encoded aspartate (D) to glutamate (E) (Supplementary Data Fig. S11). A non-synonymous replacement of C with A at 154 bp changed the encoded proline (P) to threonine (T), non-synonymous replacement of the C with T at 234 bp changed the encoded T to P, and non-synonymous replacement of the G at 288 bp with T changed the encoded E to D (Supplementary Data Fig. S11). As shown in Supplementary Data Fig. S12, the predicted isoelectric point, protein molecular weight, and 3D structure of CmCalS1 were consistent from different rootstocks. By measuring the enzyme activity of CmCalS1, the results showed that the enzyme activity of CmCalS1 in figleaf gourd was 10.33 U L^−1^, and the enzyme activity value of CmCalS1 in ‘Dongyangshenli’ was 18.61 U L^−1^, which was 1.80-fold higher that of figleaf gourd (Fig. 7A). Furthermore, CmNAC100L1 could change the enzyme activity of CmCalS1, which was manifested by a decrease in enzyme activity from 10.33 to 3.23 U L^−1^ in figleaf gourd, and an increase in enzyme activity from 18.61 to 47.78 U L^−1^ in ‘Dongyangshenli’ (Fig. 7A). Then, using the CmCalS1 sequence of figleaf gourd as a template, the mutation combination was set to verify the interconversion of D and E and the interconversion of P and T for the sites that played a key role in the change of enzyme activity. Compared with the enzyme activity of figleaf gourd, the results were divided into three categories: (i) CmCalS1 activity not detected (Supplementary Data Table S1); (ii) increased CmCalS1 activity (Fig. 7B; Supplementary Data Table S2); and (iii) decreased CmCalS1 activity (Fig. 7C; Supplementary Data Table S3). The E49D, T52P, D96E, E49D/T52P, E49D/D96E, and T52P/D96E mutants of figleaf gourd CmCalS1 showed no enzyme activity (Supplementary Data Table S1). The P57T, P57T/D96E, and E49D/T52P/P57T mutants of figleaf gourd CmCalS1 showed increased enzyme activity (Fig. 7B; Supplementary Data Table S2). The E49D/P57T, E49D/T52P/D96E, and T52P/P57T/D96E mutants of figleaf gourd CmCalS1 showed decreased enzyme activity (Fig. 7C; Supplementary Data Table S3). These results indicated that the mutation of P-57 to T-57 was responsible for the increase in activity of CmCalS1, while the other three difference points were involved in decreased activity.

## Discussion

Grafting, a widely used technique in horticultural production, can enhance yield and quality by improving the stress resistance of vegetables [27, 28]. Improving symbiotic compatibility can provide profound agronomic advantages for agricultural production. Through the analysis of molecular and physiological data, this study revealed the reasons for the incompatibility of rootstock and scion symbiosis after the survival of the grafted seedlings, and explored the molecular mechanism of the effect of Cm-miRNA164a-CmNAC100L1 module-mediated rootstock IAA regulation of callose deposition on the grafting symbiotic incompatibility reaction.

### Incompatibility of cucumber/pumpkin grafting affects the growth of grafted seedlings

Phenotype and growth parameters can intuitively reflect the level of symbiotic compatibility [8, 9]. In this study, it was clear from the analysis of the phenotype and growth parameters that graft incompatibility reactions affected plant growth (Fig. 1C–E). The PPO-oxidized phenolic substance at the grafting interface is quinine, which can defend against invasion by pathogens [29]. However, higher PPO activity, after a series of reactions, finally forms a necrotic layer and accumulates at the grafting interface, resulting in incompatibility [8]. Similarly, higher PAL activity is often accompanied by accumulation of metabolites of the phenylalanine pathway and abnormal modification of the cell wall, followed by graft incompatibility [30, 31]. Rootstock–scion interaction can also be reflected in scion leaves; incompatible combinations have a lower chlorophyll content than compatible combinations [8, 32]. In this study, we also found that PPO and PAL activities in the IG plants were the highest, and the SPAD value was the lowest, which was significantly different compared with the CG plants (Fig. 1A, B, and F), indicating that the grafting and healing process of the IG plants was intense and toxic substances accumulated at the grafting interface, which was inconducive to the healing of the wound surface and affected the material communication between rootstock and scion.

### Callose deposition at the grafting junction leads to the symbiotic incompatibility reaction of cucumber/pumpkin

Frey *et al*. [12], in a grafting study in tomato, found that there were a large number of callose deposits at the combined interface where grafting failed. Xiong *et al*. [13] also found a large amount of callose deposition at the interface of the grafted incompatible combination in melon grafting. In this study, cytological observation combined with analysis of quantitative fluorescence results also found that more callose accumulated at the interface of the IG plants (Fig. 2). Interestingly, *CmCalS1* expression patterns had a common phenomenon, which increased sharply at the 20th and 25th days after grafting, indicating that *CmCalS1* was strictly regulated in the process of callose synthesis, and mediated symbiotic incompatibility in the later stage of grafting (Fig. 2F). The accumulation of callose is regulated by co-regulation of CalS and callose hydrolase [33, 34]. The enzyme activity of CalS in the IG plants was significantly higher than that in the CG plants; it induced more accumulation of callose in the IG plants and resulted in the graft incompatibility reaction. Similarly, melon grafted onto incompatible rootstocks also induces the deposition of callose at the graft junction, which blocks the transport of photosynthate from scion to rootstock, resulting in a large amount of starch accumulated in the stem base of the scion [13].

### Cm-miR164a-CmNAC100L1 module mediates callose deposition

After grafting, rootstock and scion begin to undergo tissue adhesion, and the regeneration of vascular bundles can re-establish continuity of the transport system [35, 36]. Some members of the NAC-containing domain family promote the formation of laminar cells during grafting to participate in phloem division, and are often regarded as marker genes for vascular differentiation [23, 24]. miR164 achieves its function by negatively regulating transcription factors with NAC domains [37]. In this study, the expression of Cm-miR164a in the IG plants was higher than that in the CG plants, and the expression of the *CmNAC* gene was lower and significantly different from that in the CG plants due to the influence of Cm-miR164a targeting and endogenous regulation (Fig. 4A; Supplementary Data Fig. S6). Transient co-transformation experiments combined with 5′-RLM–RACE found that *CmNAC100L1* was the target gene of Cm-miR164a (Fig. 5). Some miRNAs and their targets have been reported to exhibit a high degree of conservation in plants [38, 39]. The sameness of the 10th and 11th bases of the miR164a mature sequence in Cucurbitaceae and the consistency of the cleavage site of *CmNAC100L1* in figleaf gourd and ‘Dongyangshenli’ indicated the conserved nature of the Cm-miR164a target (Supplementary Data Fig. S7). In the CG and IG plants, Cm-miR164a mediated the expression of *CmNAC100L1* by precise target cutting. It is worth noting that on the fifth day after grafting, compared with the IG plants, only the expression of *CmNAC100L1* in the CG plants was not affected by Cm-miR164a, and the expression did not decrease but increased (Supplementary Data Fig. S6B), suggesting that there were other regulatory modes that caused its expression to rise, and the specific regulatory mode needs further study.

Regulation of the transcription level is one of the important ways in which *CalS* genes are regulated in higher plants, such as by transcription factors, which can cause changes in *CalS* at transcription level [40, 41]. For example, auxin-induced calloplasmin-mediated intercellular filament cell gating, in which the transcript of *GLUCAN SYNTHASE LIKE 8* (*GSL8*), a gene mediating callose synthesis, is regulated by auxin-responsive factor ARF7 [42]. ARF17 regulates *GSL2* expression in the process of pollen wall formation [43, 44]. Zhou *et al*. [45] applied RNA sequencing technology to study NAC involvement in callose metabolism. In this study, we found that CmCalS1 interacted with CmNAC100L1 (Fig. 6). CmNAC100L1 was localized in the nucleus, and CmCalS1 was distributed on the cell membrane and nucleus (Supplementary Data Fig. S10), and the results of bimolecular fluorescence complementation (BiFC) showed that CmNAC100L1 interacted with CmCalS1 on the cell membrane and nucleus (Fig. 6C), suggesting that CmCalS1 might change the localization of CmNAC100L1, but this specific analysis should be further tested. Interestingly, CmNAC100L1 interacted with CmCalS1 to alter CmCalS1’s enzymatic activity (Fig. 7A). Specifically, in the compatible rootstock, the interaction between CmNAC100L1 and CmCalS1 led to a significant decrease in CmCalS1 enzyme activity (Fig. 7A). Further studies found that for CmCalS1 in compatible rootstock, E-49, T-52, and D-96 were of great significance for maintaining enzyme activity (Supplementary Data Tables S1–S3). In the incompatible rootstock, the interaction of CmNAC100L1 with CmCalS1 led to a significant increase in CmCalS1 enzyme activity (Fig. 7A), which might be the effect of T at position 57. However, the exact site of CmNAC100L1-CmCalS1 interaction is currently unknown, and further experimental verification is needed.

### Cm-miR164a-CmNAC100L1 module mediates IAA positive regulation of callose deposition at the graft junction

The death of incompatible grafted combination plants during fruit ripening is strongly associated with the large accumulation of IAA in roots, which may trigger oxidative damage and disturb the balance of IAA and cytokinins [46]. Kaseb *et al*. [47] conducted comparative transcriptome analysis of tetraploid and diploid watermelons grafted onto pumpkin rootstocks, and found that tetraploid watermelon genome replication significantly affected the transcription of genes involved in IAA signal transduction in grafted plants, resulting in higher survival rates than diploid watermelons. Fan *et al*. [48] identified 37 MeAux/IAA gene family members in cassava and found that they regulate callose development. These results indicated that IAA affects graft compatibility to a certain extent. In this study, IAA content was highly accumulated in the IG plants, and this was accompanied by higher callose content and CalS activity (Figs 2 and3A). The application of TIBA to the root of the IG plants reduced the contents of IAA and callose and CalS activity in the rootstock at the graft junction (Fig. 3E–G). miR164 has been shown to be involved in organ formation, and auxin signaling in the meristem [49]. Here, it was found that IAA negatively regulated the expression of Cm-miR164a (Fig. 4C and D). Therefore, IAA regulated the expression of *CmNAC100L1* by negatively regulating the expression of Cm-miR164a, which in turn affected the activity of CmCalS1 and the deposition of callose.

**Figure 8:**
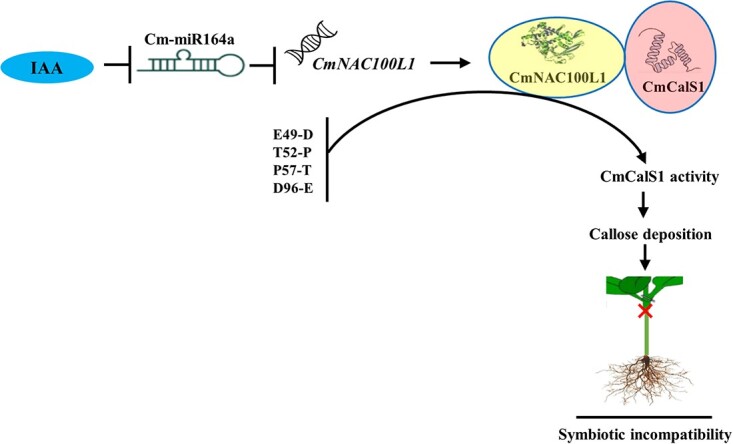
A proposed model for the IAA-regulated molecular pathway of symbiotic incompatibility of cucumber/pumpkin grafted seedlings. IAA achieved positive regulation of *CmNAC100L1* by negatively regulating the expression of Cm-miR164a. Due to the difference in *CmCalS1* bases in the compatible combination and incompatible combination (IG), CmNAC100L1 interacted with CmCalS1 in the IG to increase its activity, which promoted the accumulation of callose deposition at the graft union, resulting in a symbiotic incompatibility reaction.

Taking our results together, we provided a molecular regulatory pathway for the symbiotic incompatibility response of cucumber/pumpkin grafted seedlings regulated by IAA (Fig. 8). IAA regulated the expression of *CmNAC100L1* by negatively regulating the expression of Cm-miR164a, and the interaction between CmNAC100L1 and CmCalS1 improved enzyme activity of CmCalS1 in the incompatible combination, which promoted a large amount of callose deposition at the graft union, leading to a symbiotic incompatibility reaction.

## Materials and methods

### Plant materials

In this study, cucumber (Jinchun No. 4) was selected as the scion, and two pumpkin varieties (figleaf gourd, *Cucurbita ficifolia* Bouche., compatible rootstock) and (‘Dongyangshenli’, *Cucurbita moschata* D., incompatible rootstock) with different symbiotic compatibility were selected as the rootstocks [8]. The rootstock pumpkin seeds were soaked in distilled water for 8 h at room temperature and then placed at 28°C for 48 h. The scion cucumber seeds were soaked for 4 h and then placed in a germination box at 28°C for 24 h. Pumpkin seeds as rootstocks were sown in a 15-hole tray (540 × 280 × 60 mm), which was filled with a substrate of vinegar residue:peat:vermiculite = 2:1:1 (v:v:v). Cucumber seeds used as scions were sown 3 days after the rootstock seeds. When the scion cotyledons and the first true leaf of the rootstocks were fully expanded, the split grafting method was used for grafting.

The experiment was set up with four treatments: (i) NG as a control; (ii) SG as another control; (iii) CG, cucumber/figleaf gourd as the compatible combination; and (iv) IG, cucumber/‘Dongyangshenli’ as the incompatible combination. After grafting, the plants were placed in an artificial growth chamber (Aessense, Shanghai, China) for 7 days to heal the grafting wounds. The humidity in the chamber was kept between 85 and 100%, and the temperature was maintained at 25°C.

### Root IAA or TIBA treatment

Nineteen days after grafting, the CG plants were cultured in 1/2 Hoagland nutrient solution containing 0 or 10 μM IAA, and the IG plants were cultured in 1/2 Hoagland nutrient solution containing 0 or 10 μM TIBA. After IAA or TIBA treatment for 6 days, the grafted healing part was cut with scissors, and the bonded scion part was quickly removed, leaving ~0.5 cm of the rootstock part to determine IAA and callose contents, and the activities of CalS and callose hydrolase. For the NG plants, cucumber seedlings with the same growth as the grafted plants were selected, and 0.5 cm of the stem segment at the same height was harvested for analysis (Supplementary Data Fig. S13).

### Leaf IAA treatment

To analyze the role of IAA on the expression of Cm-miR164a in ‘Dongyangshenli’, plants with two expanded leaves were sprayed with 100 μM IAA, and the first leaves from top to bottom were taken at 0, 3, 6, 12, and 24 h to determine the Cm-miR164a expression level.

### Measurement of IAA content

On the 25th day after grafting, IAA content was determined in the rootstock of the grafted healing site after removing the scion. The IAA content was determined using an ELISA kit (MM-0953O1, Jiangsu Meimian Industrial Co., Ltd, Yancheng, China). Three replicates were tested per sample, and the experiment was performed three times.

### Determination of SPAD index

The SPAD index was determined in the third true leaves from top to bottom using a SPAD 502 Plus portable chlorophyll meter (Konica Minolta Optics Co., Ltd, Osaka, Japan) 25 days after grafting.

### Determination of growth parameters of grafted seedlings

The fresh and dry weights of the whole grafted plants were measured according to the method described previously [9].

### Measurement of polyphenol oxidase and phenylalanine ammonialyase activity

Twenty-five days after grafting, 0.2 g of the stem segment of the grafted healing site was taken to determine the activity of PAL as described previously [50]. The reaction solutions were 0.2 ml of 40 mM phenylalanine, 0.4 ml of 100 mM Tris–HCl (pH 8.8), and 0.2 ml of enzyme extract; the preparation was incubated at 37°C for 30 min and the reaction was stopped by adding 25% trichloroacetic acid. Then, the change in OD_280_ value in 2 min was determined and a decrease in OD_280_ of 0.001 per minute was taken as 1 U of enzyme activity. PPO activity was determined by adding 50 μl of 60 mM catechol, 2.85 ml of 50 mM PBS (pH 7.0), and 0.1 ml of supernatant. The change in OD_390_ within 2 min was measured [51].

### Measurement of callose content, callose synthase and hydrolase activity

Twenty-five days after grafting, stem segments (0.3 g) of the rootstock in the grafted junction were ground in phosphate buffer (pH 7.3, Solarbio, Beijing, China), and the homogenate was centrifuged for 15 min at 4°C, 4000 rpm. The supernatant was used to measure the callose content and CalS and hydrolase activities. The content of callose was determined using a callose content assay kit (MM-35931O1, Jiangsu Meimian Industrial Co., Ltd). The activity of CalS was determined using the CalS activity assay kit (MM-2540O1, Jiangsu Meimian Industrial Co., Ltd), and the activity of callose hydrolase was determined using a callose hydrolase activity kit (MM-63294O1, Jiangsu Meimian industrial Co., Ltd) according to the instructions of the kits.

### Cytological observation of callose deposition at the graft junction

The stem segment at the junction of the rootstock and scion was quickly cut with a blade 25 days after grafting and placed in FAA fixative solution. The stem segment was completely wrapped with tissue freeze glue and left for 2 h. Then, the sample was cut into 10-μm vertical sections, and each section was placed on a slide at room temperature to allow the tissue freeze glue to solidify slightly. The section was immersed in 96% ethanol for 6 h, the ethanol was left to dry, and histochemical staining was performed as previously described [52]. The deposition site of callose at the graft junction was observed and photographed with a BX51 fluorescence microscope (Olympus, Tokyo, Japan). The area of callose deposition in a single vascular bundle was measured using CaseViewer software (3DHistech, Budapest, Hungary).

### Cm-miR164a target gene prediction

The binding sites of Cm-miR164a and its target genes were predicted using psRNATarget (https://www.zhaolab.org/psRNATarget/) [53]. The target genes with the highest probability were selected by parameters such as expectation and UPE value.

### 5′-RLM–RACE

To analyze the cleavage relationship of Cm-miR164a to its target gene, 5′-RLM–RACE was performed according to the instructions of the FirstChoice™ RLM–RACE kit (AM1700, Invitrogen, Carlsbad, CA, USA). The primers used are shown in Supplementary Data Table S4.

### Dual luciferase assay

The dual luciferase assay was performed as described by Yang *et al*. [54]. The 395-bp sequence of *CmNAC100L1* containing the Cm-miR164a predicted binding site was ligated into the pAC006 vector. The 115-bp precursor sequence of Cm-miR164a was synthesized by General Biosystems (Anhui) Co., Ltd (Chuzhou, China), and was inserted into the pCAMBIA1301 vector, and then transformed into EHA105 (CC96314, Tolobio, Shanghai, China). Forty-eight hours after *Agrobacterium tumefaciens* injection, tobacco leaves were photographed with a fluorescence detection imager (5200Multi, Tanon, Shanghai, China).

### RNA isolation and qPCR analysis

Total miRNA and RNA were isolated using the miRcute miRNA extraction kit (DP501, Tiangen, Beijing, China) and an RNAsimple Total RNA kit (DP419, Tiangen), respectively, from all collected samples. The expression level of the gene was detected with a ChamQ SYBR qPCR Master Mix Kit (Q311-02, Vazyme) using the primers shown in Supplementary Data Table S5. *U6*, *TIP4A*, and *18S* were selected as the internal reference.

### GUS histochemical staining analysis

The CDS of *CmNAC100L1* was amplified with specific primers (Supplementary Data Table S6) and inserted into the pBI121 vector. The designed synonymous mutant *Cmm5NAC100L1* sequence was sent to General Biosystems (Anhui) Co., Ltd for artificial synthesis and constructed into the pBI121 vector. The promoter sequence of *Cm-MIR164a* obtained by NCBI was sent to General Biosystems (Anhui) Co., Ltd for artificial synthesis, and the *CaMV35S* promoter in the pBI121 vector was replaced with the promoter of *Cm-MIR164a*. Tobacco transient transformation was performed as described by Wang *et al*. [55]. In order to analyze the effect of IAA on the expression of Cm-miR164a, tobacco leaves were sprayed with 100 μM IAA 1 day after injection. Tobacco leaves (0.1 g) were taken for GUS enzyme activity detection with a GUS gene quantitative detection kit (SL7161, Coolaber, Beijing, China) 2 days after injection.

### Expression and purification of recombinant protein

The CDS sequences of figleaf gourd and ‘Dongyangshenli’ *CmNAC100L1* and *CmCalS1* were amplified and inserted into the pET32a vector. They were transformed into *Escherichia coli* Rosetta DE3 (CC96185, Tolobio). The bacteria were cultured in LB medium with ampicillin antibiotics at 37°C. When the OD value reached 0.6–0.8, IPTG (0.3 mM) was added to the medium, which was shaken at 25°C for 4 h. Then, the bacteria were centrifuged at 4°C, 12 000 rpm for 10 min, and the pellet was suspended with pre-cooled PBS containing 1 mM PMSF and 1 mg ml^−1^ lysozyme (12650-88-3, Solarbio). The suspension was used for ultrasonic crushing with an ultrasonic crusher (FB705220, Fisher 705, Waltham, MA, USA). The recombinant proteins were purified with a His-tagged protein purification kit (P2229S, Beyotime, Shanghai, China).

### Bimolecular fluorescence complementation assay

A BiFC experiment was conducted as described previously [56]. The CDSs of *CmNAC100L1* and *CmCalS1* from ‘Dongyangshenli’ were amplified to construct *CmNAC100L1*-YFP^c^ and *CmCalS1*-YFP^n^ vectors, and positive plasmids were transferred into *A. tumefaciens* strain GV3101. Forty-eight hours after infection, YFP fluorescence was observed using a laser confocal microscope (LSM 800, Zeiss, Oberkochen, Germany).

### Pull-down experiment

The CDS of *CmCalS1* was amplified by PCR using gene-specific primers (Supplementary Data Table S6) and ligated into the pGEX4T-1 vector. Then, the recombinant protein was expressed according to the above method. The pull-down experiment was carried out according to the instructions of the GST pull-down assay kit (P2251, Beyotime). The proteins were analyzed as previously described [57]. A mouse anti-GST antibody (MA181040, Pierce, USA) and a mouse anti-His antibody (MA121315, Pierce, USA) were used.

### Firefly luciferase complementation

The CDSs of *CmNAC100L1* and *CmCalS1* were amplified by PCR using gene-specific primers (Supplementary Data Table S6) and ligated into the pCAMBIA1301-nLUC and pCAMBIA1301-cLUC vectors, respectively. The firefly luciferase complementation experiment was conducted as described by Hou *et al*. [58].

### Subcellular localization

Subcellular localization of CmNAC100L1 and CmCalS1 was performed as described earlier [1]. The full-length CDSs of *CmNAC100L1* and *CmCalS1* were ligated into pAC402-GFP vector to generate *CmNAC100L1*-GFP and *CmCalS1*-GFP fusion expression vectors. Subsequently, pAC402-*CmNAC100L1*-GFP, pAC402-*CmCalS1*-GFP, and pAC402-GFP empty carriers were transformed into *A. tumefaciens* strain GV3101 and infiltrated into the leaves of tobacco leaves expressing H2B-RFP as markers for nuclei [59]. Forty-eight hours after inoculation, fluorescence signals were detected with an LSM800 confocal microscopy (Zeiss, Germany).

### Construction of site-directed mutation vector

The figleaf gourd *CmCalS1* sequence was used as the template to design the mutation sequence, which was sent to General Biosystems (Anhui) Co., Ltd for artificial synthesis, and was inserted into the pET32a vector. Protein purification and enzyme activity determination were performed as described above.

### Analysis of gene sequence, amino acid sequence, and *cis*-acting element of promoter

The gene sequence, protein isoelectric point, and molecular weight were calculated using BioXM2.7.1 software, and the 3D structure of CmCalS1 protein was drawn using the online website (http://missense3d.bc.ic.ac.uk/missense3d/). The promoter *cis*-acting element of *Cm-MIR164a* was analyzed using PlantCARE and PLACE.

### Yeast one-hybrid assay

The yeast one-hybrid assay was carried out referring to the method of Wang *et al*. [60]. The *CmCalS1* promoter sequence with a length of 1056 bp was cloned, and constructed into the pAbAi vector. The constructed pAbAi-*CmCalS1* was digested by BstBI and transferred into Y1H yeast. The CDS of *CmNAC100L1* was amplified with specific primers (Supplementary Data Table S6) and constructed into the pGADT7 vector. The pGADT7-*CmNAC100L1* recombinant vector was transformed into yeast cells harboring the *CmCalS1* promoter, and grown on selection medium with 300 ng ml^−1^ aureobasidin A.

### Statistical analysis

Twelve grafted seedlings with the same growth trend were randomly selected for the test. All the indexes were measured at least three times with three biological replicates. All data were represented by the mean ± standard deviation (*n* = 3). SPSS26.0 statistical software (IBM SPSS statistics) was used for analysis of variance. Differences between treatments were detected at *P* < 0.05 level by Tukey’s test.

## Supplementary Material

Web_Material_uhad287

## Data Availability

The data supporting the findings of this study are available within the paper and its supplementary information files.
