# Supplementary material for: IAA-miR164a-NAC100L1 module mediates symbiotic incompatibility of cucumber/pumpkin grafted seedlings through regulating callose deposition
Source: Hortic Res. 2023 Dec 29;11(2):uhad287. doi: 10.1093/hr/uhad287 (PMC10873582; doi:10.1093/hr/uhad287)
Supplement: Web_Material_uhad287 [file web_material_uhad287.zip › Supplementary Figures-20231128.pdf]

Figure S1

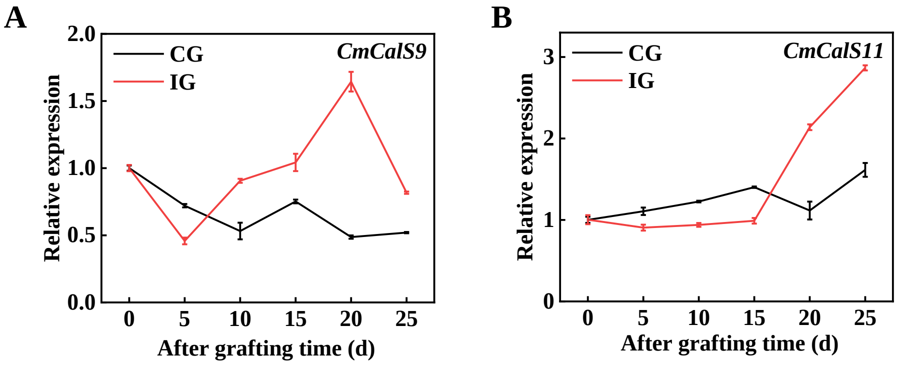

Figure S1. The expression of *CmCalS9* and *CmCalS11* in the compatible combination (CG) and incompatible combination (IG) after grafting. **A** *CmCalS9* expression. **B** *CmCalS11* expression. The expression of *CmCalS9* and *CmCalS11* was analyzed by qPCR in the rootstock of the grafted junction of different compatible combinations after grafting. The data represent the mean ± SD (n=3). According to the Tukey's test, means with the same letter did not differ significantly at $P<0.05$.

Figure S2

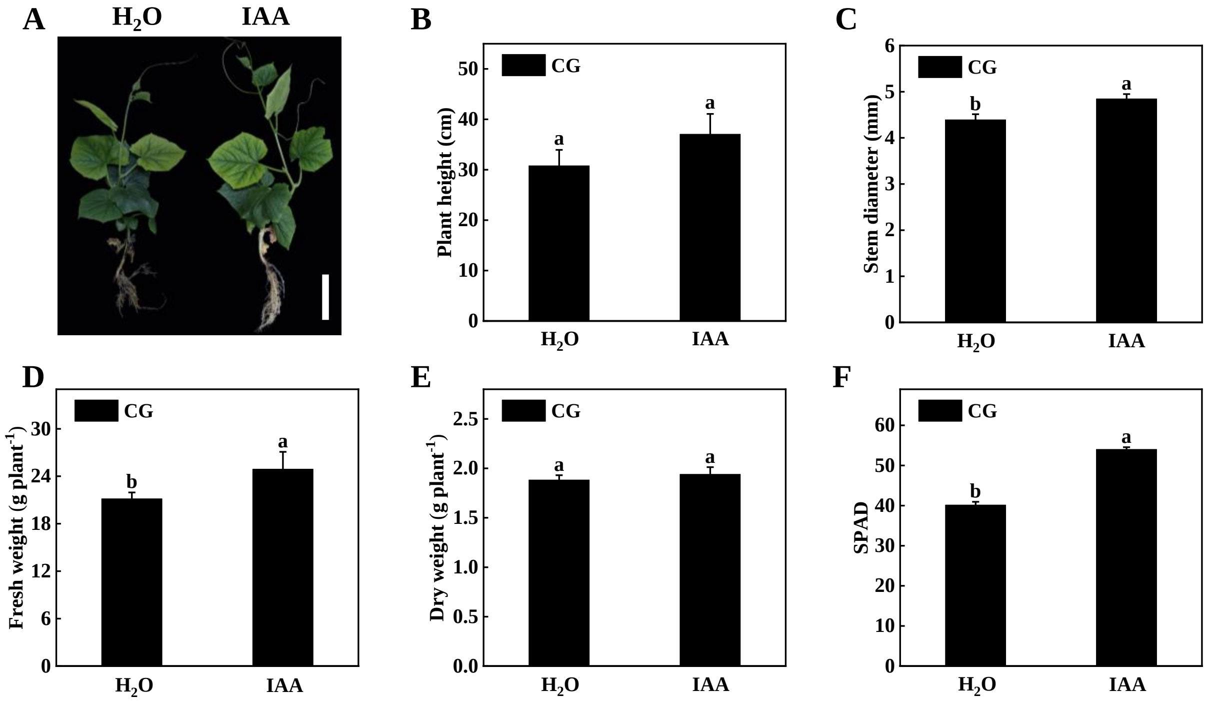

Figure S2. Effects of exogenous treatment with IAA in the root of compatible combination (CG) on the growth of grafted plants. **A** Plant phenotype after IAA treatment for 6 d. **B** Plant height. **C** Stem diameter. **D** Fresh weight. **E** Dry weight. **F** SPAD. After grafting for 19 d, 10 μM IAA was treated and the phenotype and growth parameters were measured after treatment for 6 d. The data represent the mean ± SD (n=3). According to the Tukey's test, means with the same letter did not differ significantly at *P*<0.05. Bar: 5 cm.

Figure S3

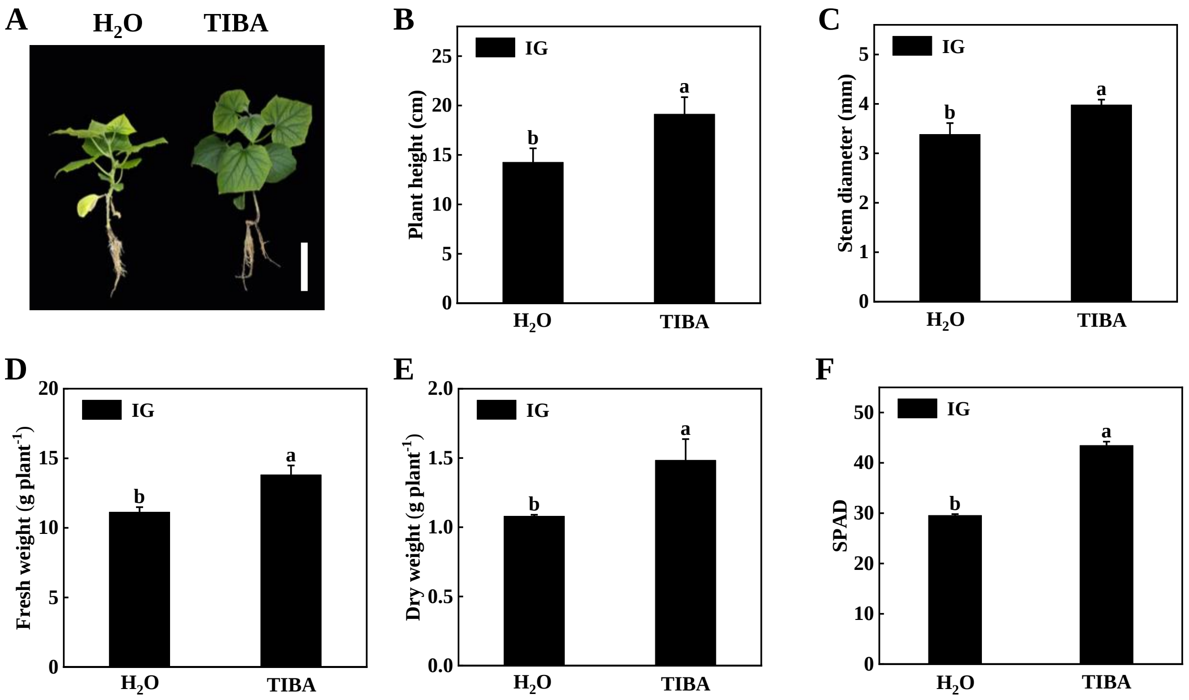

Figure S3. Effects of exogenous treatment with TIBA in the root of incompatible combination (IG) on the growth of grafted plants. **A** Plant phenotype after TIBA treatment for 6 d. **B** Plant height. **C** Stem diameter. **D** Fresh weight. **E** Dry weight. **F** SPAD. After grafting for 19 d, 10 μM TIBA was treated and the phenotype and growth parameters were measured after treatment for 6 d. The data represent the mean ± SD (n=3). According to the Tukey's test, means with the same letter did not differ significantly at $P<0.05$. Bar: 5 cm.

Figure S4

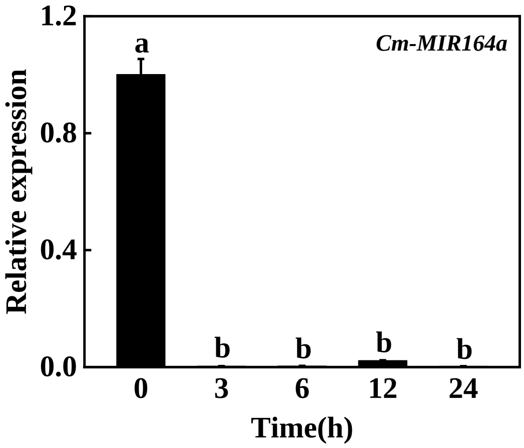

Figure S4. Expression levels of Cm-miR164a after IAA treatment. The expression of *Cm-MIR164a* was analyzed by qPCR in the leaves of Dongyangshenli after IAA treatment at the indicated time points. The data represent the mean ± SD (n=3). According to the Tukey's test, means with the same letter did not differ significantly at $P<0.05$.

Figure S5

A

Expect	UPE	Alignment
2.0	9.221	miRNA 21 UCGUGCACGGGACGAAGAGGU 1<br>::::::::::::::::::<br>Target 617 CUCACGUGCCCUGCUUCUCCA 637

B

Expect	UPE	Alignment
9.5	20.009	miRNA 21 UCGUGCACGGGACGAAGAGGU 1<br>: :.::::::.:.:<br>Target 424 GCCCAAUCCUCUGCUUUUUCC 444

C

Expect	UPE	Alignment
3.0	15.68	miRNA 21 UCGUGCACGGGACGAAGAGGU 1<br>::::::::::::::::::<br>Target 656 AUAACGUGCCCUGCUUCUCCA 676
3.0	15.68	miRNA 21 UCGUGCACGGGACGAAGAGGU 1<br>::::::::::::::::::<br>Target 1754 AUAACGUGCCCUGCUUCUCCA 1774

Figure S5. psRNATarget online prediction the target gene of Cm-miR164a. **A** The target values of Cm-miR164a and *CmNAC100L1*. **B** The target values of Cm-miR164a and *CmNAC79*. **C** The target values of Cm-miR164a and *CmNAC100L2*.

Figure S6

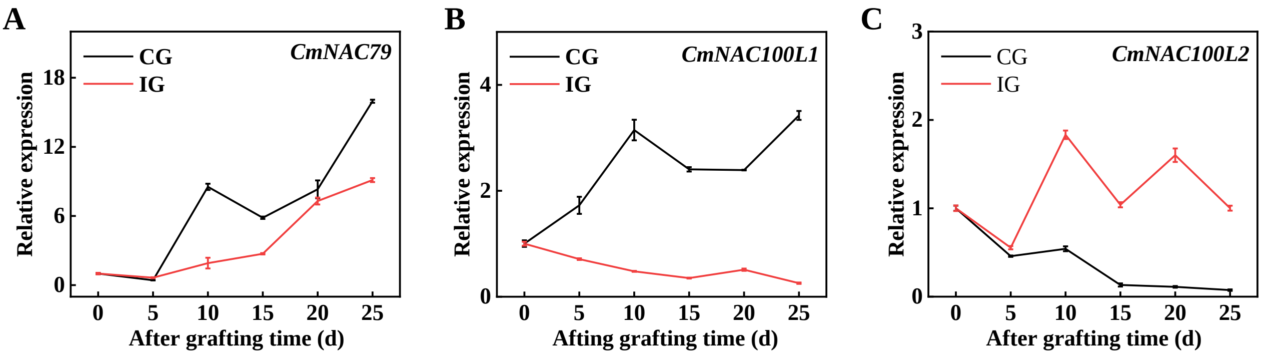

Figure S6. The expression of *CmNAC79*, *CmNAC100L1* and *CmNAC100L2* in the compatible combination (CG) and incompatible combination (IG) after grafting. **A** *CmNAC79* expression. **B** The expression of *CmNAC100L1*. **C** *CmNAC100L2* expression. The expression of *CmNAC79*, *CmNAC100L1* and *CmNAC100L2* was analyzed by qPCR in the rootstock of the grafted junction of different compatible combinations after grafting. The data represent the mean ± SD (n=3). According to the Tukey's test, means with the same letter did not differ significantly at $P<0.05$.

Figure S7

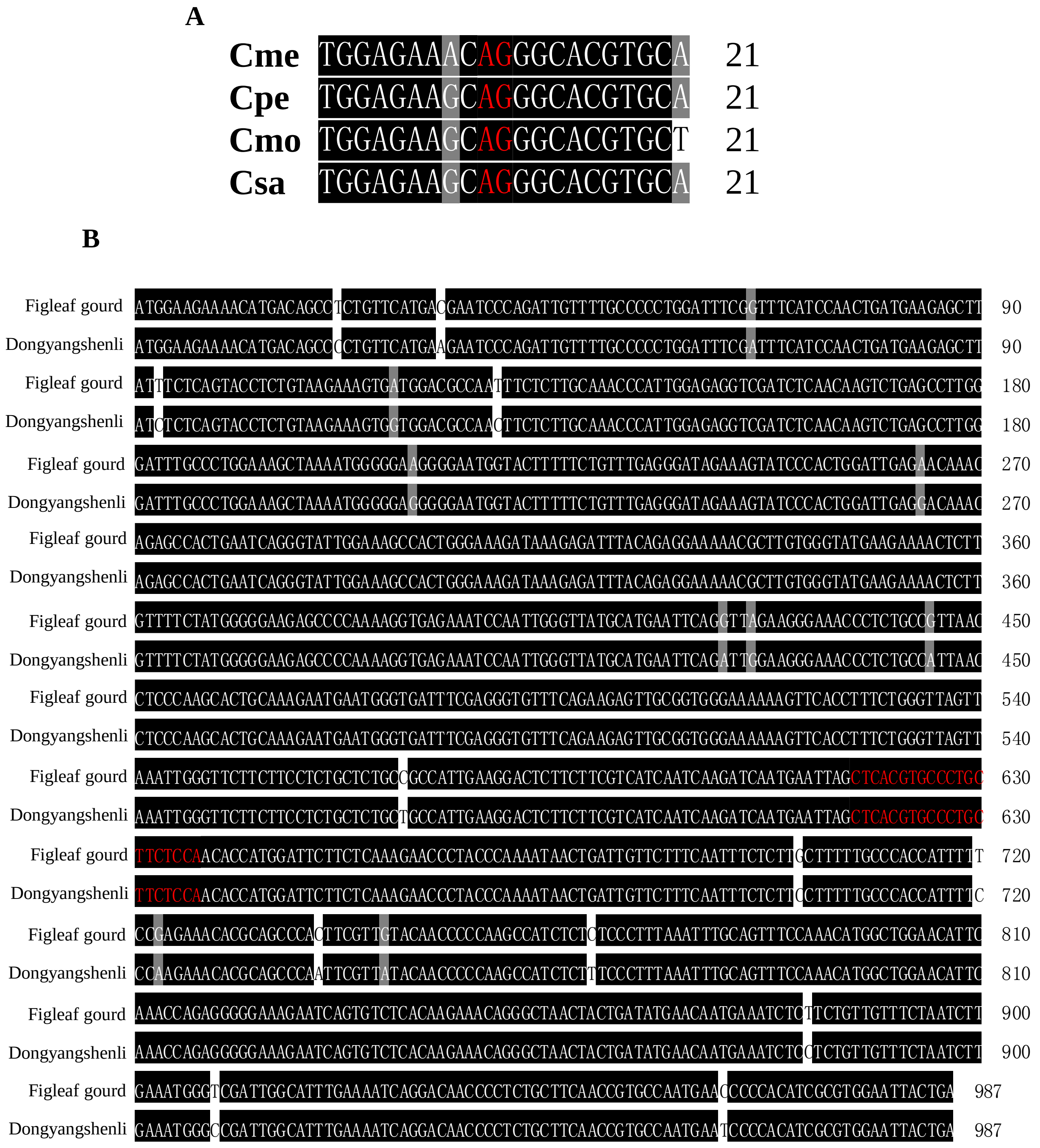

Figure S7. Sequence alignment of miR164a in *Cucurbitaceae* species and *CmNAC100L1* CDS sequence. **A** Sequence alignment of Cm-miR164a in *Cucurbitaceae* species. Cme, *Cucumis melo*; Cpe, *Cucurbita pepo*; Cmo, *Cucurbita moschata*; Csa, *Cucumis sativus*. **B** *CmNAC100L1* CDS sequence alignment in the compatible rootstock (figleaf gourd) and incompatible rootstock (Dongyangshenli).

Figure S8

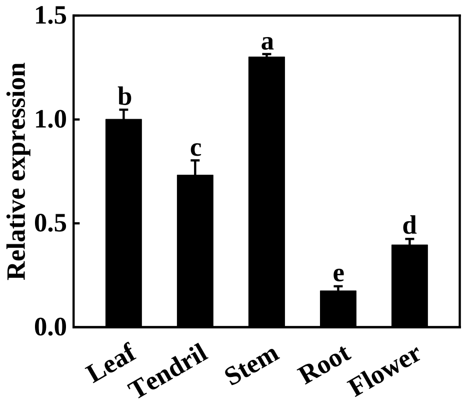

Figure S8. Analysis of the expression pattern of *CmNAC100L1* in different tissues of Dongyangshenli. The data represent the mean ± SD (n=3). According to the Tukey's test, means with the same letter did not differ significantly at $P<0.05$.

Figure S9

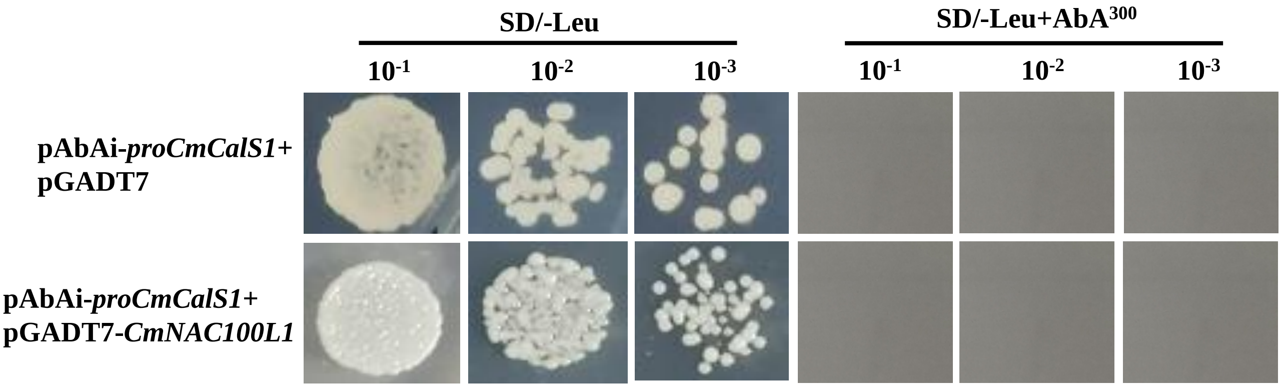

Figure S9. Yeast one-hybrid assay indicating CmNAC100L1 failed to bind to the promoter of *CmCalS1*.

Figure S10

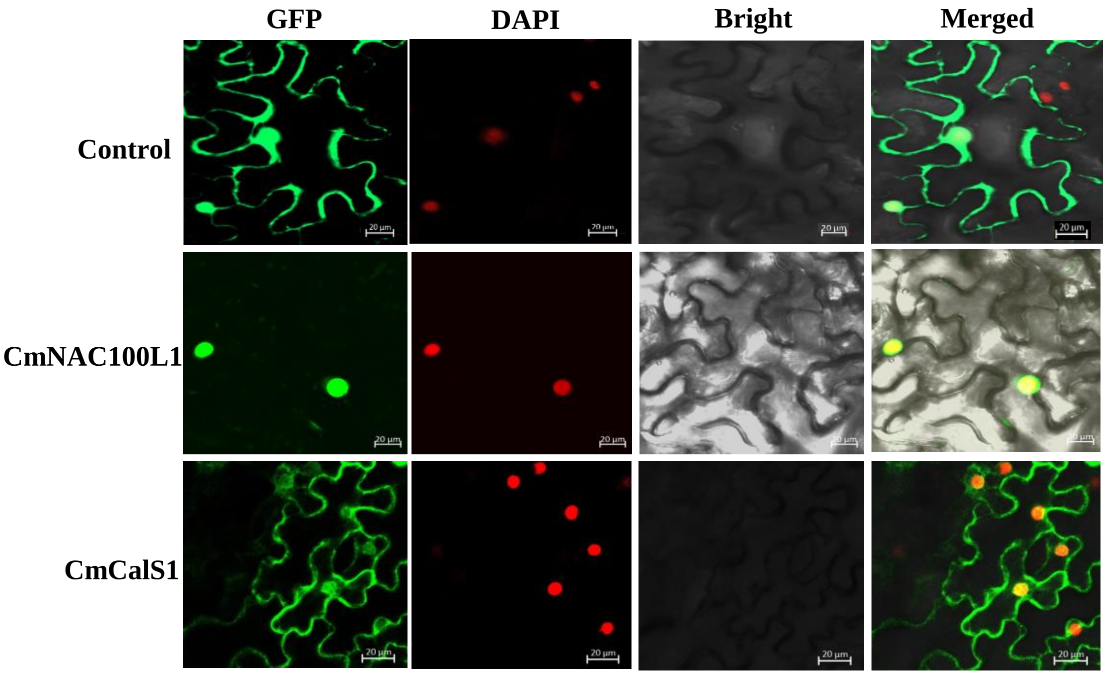

Figure S10. CmCalS1 and CmNAC100L1 subcellular localization in tobacco cells. Bar: 20 µm.

Figure S11

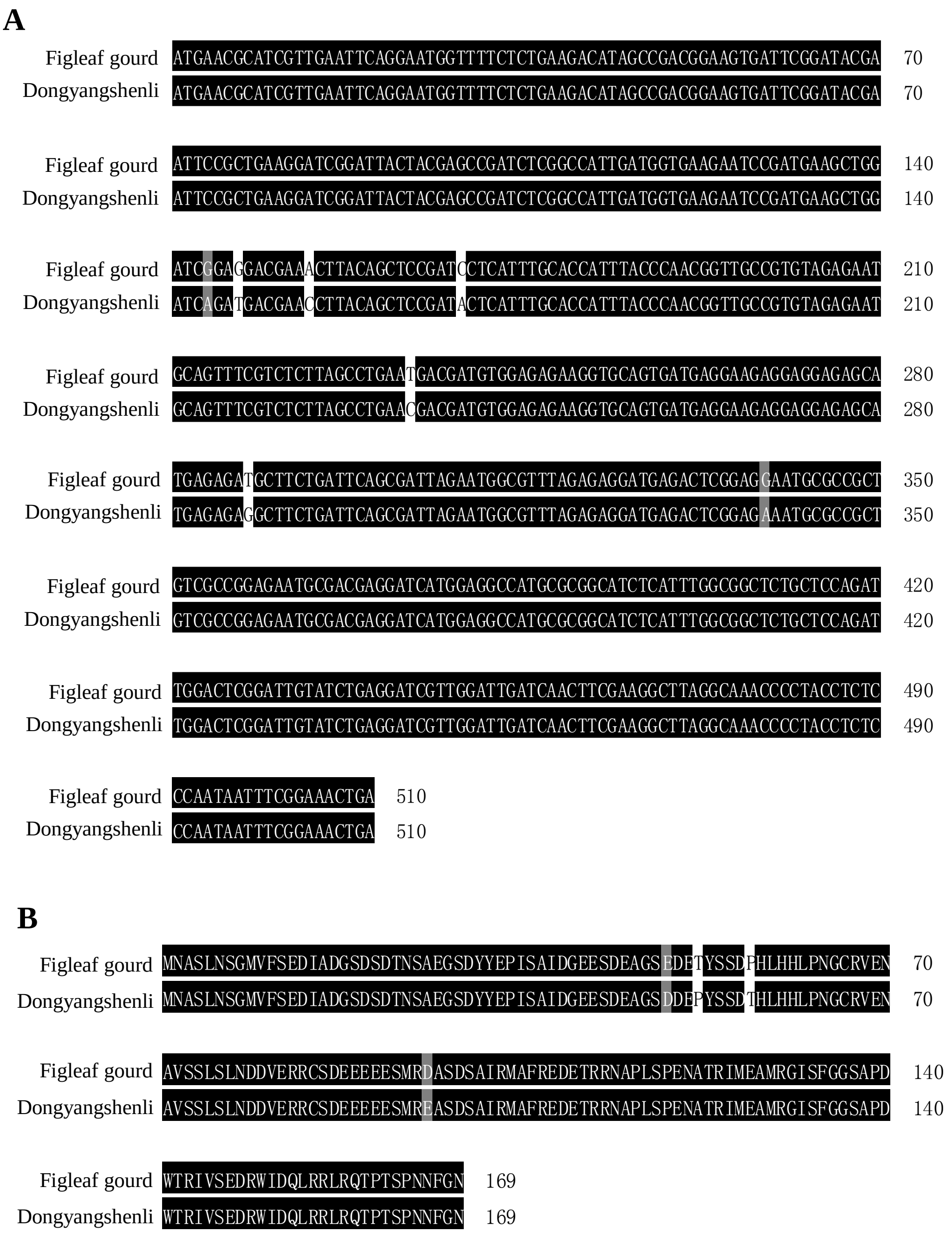

Figure S11. Sequence alignment of CmCalS1 in the compatible rootstock (figleaf gourd) and incompatible rootstock (Dongyangshenli). **A** *CmCalS1* CDS sequence alignment in Figleaf gourd and Dongyangshenli. **B** CmCalS1 protein sequence alignment in Figleaf gourd and Dongyangshenli.

Figure S12

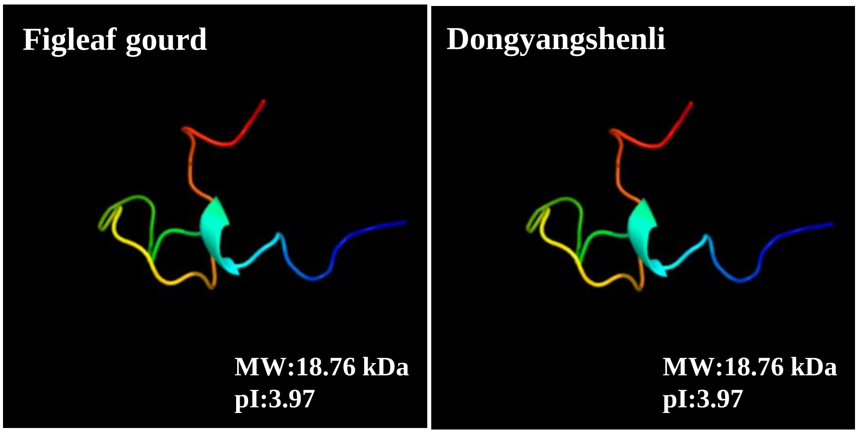

Figure S12. Comparison of isoelectric point, 3D structure, and molecular weight of CmCalS1 in figleaf gourd and Dongyangshenli. MW, molecular weight; pI, isoelectric point.

Figure S13

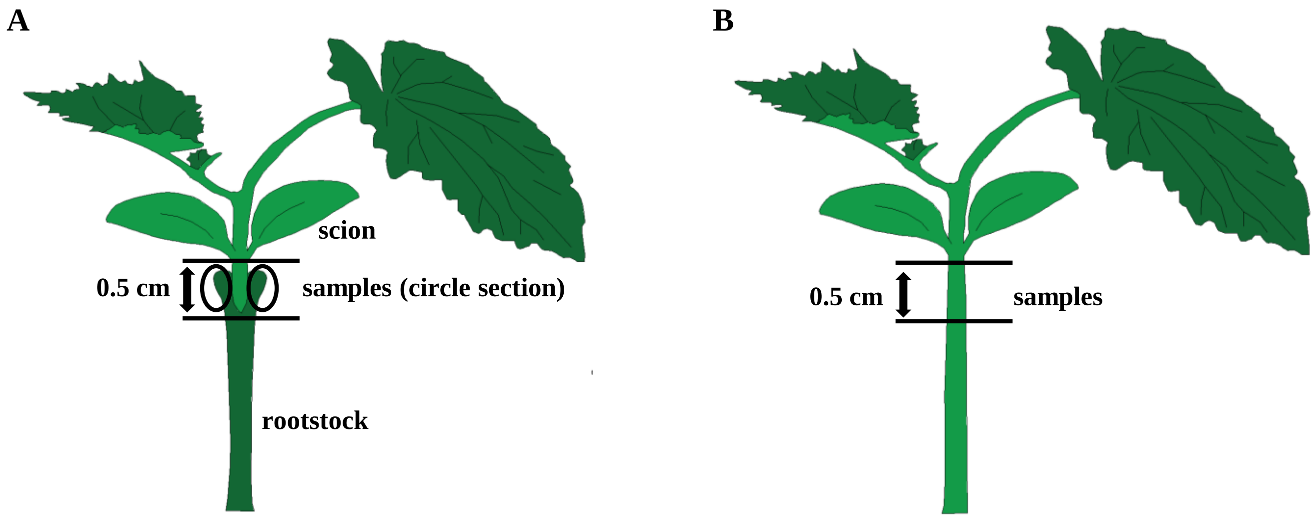

Figure S13. Schematic diagram of harvesting the samples. **A** Schematic diagram of harvesting the samples in grafted plants. The grafted healing part was cut with scissors, quickly removed the bonded scion part, and left about 0.5 cm of rootstock part (circle section) for sample. **B** Schematic diagram of harvesting the samples in non-grafted plants. Cucumber seedlings with the same growth as the grafted plants were selected, and 0.5 cm of the stem segment at the same height was harvest.
