# Supplementary material for: IAA-miR164a-NAC100L1 module mediates symbiotic incompatibility of cucumber/pumpkin grafted seedlings through regulating callose deposition
Source: Hortic Res. 2023 Dec 29;11(2):uhad287. doi: 10.1093/hr/uhad287 (PMC10873582; doi:10.1093/hr/uhad287)
Supplement: Web_Material_uhad287 [file web_material_uhad287.zip › Supplementary Tables-20231128.docx]

**Table S1.** Mutation of figleaf gourd CmCalS1without detected the enzyme activity.

| Position | IG-CalS1 | CG-CalS1 | Mutation sequence 1 | Mutation sequence 2 | Mutation sequence 3 | Mutation sequence 4 | Mutation sequence 5 | Mutation sequence 6 | Mutation sequence 7 |
| --- | --- | --- | --- | --- | --- | --- | --- | --- | --- |
| 49 | D | E | D | E | E | D | D | E | E |
| 52 | P | T | T | P | T | P | T | P | P |
| 57 | T | P | P | P | P | P | P | T | P |
| 96 | E | D | D | D | E | D | E | D | E |
| Enzyme activity | 18.61 | 10.33 | nd | nd | nd | nd | nd | nd | nd |

**Table S2.** Mutation of figleaf gourd CmCalS1 increased the enzyme activity.

| Position | IG-CalS1 | CG-CalS1 | Mutation sequence 11 | Mutation sequence 12 | Mutation sequence 13 |
| --- | --- | --- | --- | --- | --- |
| 49 | D | E | E | E | D |
| 52 | P | T | T | T | P |
| 57 | T | P | T | T | T |
| 96 | E | D | D | E | D |
| Enzyme activity | 18.61 | 10.33 | 32.74 | 28.89 | 17.53 |

**Table S3.** Mutation of figleaf gourd CmCalS1decreased the enzyme activity.

| Position | IG-CalS1 | CG-CalS1 | Mutation sequence 8 | Mutation sequence 9 | Mutation sequence 10 |
| --- | --- | --- | --- | --- | --- |
| 49 | D | E | D | D | E |
| 52 | P | T | T | P | P |
| 57 | T | P | T | P | T |
| 96 | E | D | D | E | E |
| Enzyme activity | 18.61 | 10.33 | 5.82 | 3.13 | 3.85 |

**Table S4.** Primer sequences used for 5' RLM-RACE.

| Target gene | Outer primer (5’-3’) | Inner primer (5’-3’) |
| --- | --- | --- |
| *CmNAC100L1* | GGTTGAAGCAGAGGGGTTGT | GTTTCTCGGAAAAATGGTGGGCAAA |
| *CmNAC100L2* | TGTTGACATCGGTGGTGAGG | TTTCCGCCACTGACGTTCTGAAT |
| *CmNAC79* | GGAGGCGATGGATGGTTGTT | AGGCGATGGATGGTTGTTAGCC |

**Table S5.** Primer sequences used for qPCR in pumpkin.

| Gene name | Forward primer | Reverse prime |
| --- | --- | --- |
| *CmCalS1* | TCATTTGCACCATTTACCC | CTCCTCCTCTTCCTCATCAC |
| *CmCalS9* | ATTTACGGGCAGTGTTTC | ATGTTGATAGCACGGGAG |
| *CmCalS11* | AGGTGGCTCTTGACTACGC | CCATTACTTGGGTCTGTGC |
| *CmNAC79* | GGAGGTGTCTTGTTGGGTTCA | GCAGAGGATTGGGCGTGT |
| *CmNAC100L1* | TTCTTCTTCCTCTGCTCTG | TCTTGTGAGACACTGATTCT |
| *CmNAC100L2* | CTACAGGGAAAGATAAAGACA | CTGCAAATCACCCACTCA |
| *TIP4A* | TGGGAGGATTGCGAGGAGA | AAGTGATATGCCATTGTCAGC |
| *18S* | AAACCTTACCAGCCCTTGAC | CGCTCGTTATAGGACTTGACC |
| *U6* | GGGGACATCCGATAAAATT | ATCCCTTCCACAGATTGCTT |

Table S6. Primer sequences used for vector construction.

| Gene name | Forward primer (5’-3’) | Reverse prime (5’-3’) |
| --- | --- | --- |
| pac006-CmNAC100L1-395 | ggaaagatcgccgtgggatccTAACAGGTTAGAAGGGAAACCCTC | actctagggactagtcccgggTGAATGTTCCAGCCATGTTTGG |
| Pac402-CmNAC100L1 | tacaaatctatctctctcgagATGGAAGAAAACATGACAGCCTC | ggatccccgggtaccgagctcGTAATTCCACGCGATGTGGG |
| Pac402-CmCalS1 | tacaaatctatctctctcgagATGAACGCATCGTTGAATTCAG | ggatccccgggtaccgagctcGTTTCCGAAATTATTGGGAGAGG |
| pAbAi-CmCalS1 | cttgaattcgagctcggtaccGCAGTGCCTTGGCTAGCATC | atacagagcacatgcctcgagTTGTTGGCAATAACGCGAATC |
| pGADT7-CmNAC100L1 | gccatggaggccagtgaattcATGGAAGAAAACATGACAGCCTC | cagctcgagctcgatggatccTCAGTAATTCCACGCGATGTGG |
| PBI121-CmNAC100L1 | gagaacacgggggactctagaATGGAAGAAAACATGACAGCCTC | ataagggactgaccacccgggGTAATTCCACGCGATGTGGG |
| Pet32a-CmNAC100L1 | gccatggctgatatcggatccATGGAAGAAAACATGACAGCCTC | gcaagcttgtcgacggagctcTCAGTAATTCCACGCGATGTGG |
| PGEX4T1-CmCalS1 | gatctggttccgcgtggatccATGAACGCATCGTTGAATTCAG | ctcgagtcgacccgggaattcTCAGTTTCCGAAATTATTGGGAG |
| CmCalS1-YFP^n^ | atttacgaacgatagttaattaaATGAACGCATCGTTGAATTCAG | actgccacctcctccactagtGTTTCCGAAATTATTGGGAGAGG |
| CmNAC100L1-YFP^C^ | atttacgaacgatagttaattaaATGGAAGAAAACATGACAGCCTC | actgccacctcctccactagtGTAATTCCACGCGATGTGGG |
| cluc- CmCalS1 | tacgcgtcccggggcggtaccATGAACGCATCGTTGAATTCAG | cagtcgacgcgttgtggatccTCAGTTTCCGAAATTATTGGGAG |
| nluc- CmNAC100L1 | acgggggacgagctcggtaccATGGAAGAAAACATGACAGCCTC | cagtcgacgcgttgtggatccGTAATTCCACGCGATGTGGG |
